# Killing Two Birds with One Stone? Green Dead Ends and Ways Out of the COVID-19 Crisis

**DOI:** 10.1007/s10640-020-00443-y

**Published:** 2020-07-08

**Authors:** Erik Gawel, Paul Lehmann

**Affiliations:** 1 Helmholtz Centre for Environmental Research – UFZ, Permoserstr. 15, 04318 Leipzig, Germany; 2 University of Leipzig, Ritterstr. 12, 04109 Leipzig, Germany

**Keywords:** Climate change, Coronavirus, Recovery, Subsidies

## Abstract

The coronavirus crisis has opened up a window of opportunity for transformation. This should be used without getting off the regulatory track. Green recovery programs must not be reduced to a mere competition for green subsidies. Abandoning barriers to green investments and imposing a carbon price are equally important. Where economically sensible, green subsidies should contribute both to stabilizing the economy and mitigating climate change. Moreover, smart green recovery programs may contribute to raising revenues for the additionally necessary public expenditures.

Public measures to combat the COVID-19 pandemic have led to a severe economic crisis. In order to cope with this crisis, comprehensive government aid is being requested. Accordingly, governments across the world have pledged billions of Euros for extensive recovery programs. One of the main questions debated in this context at the moment is how “green” these recovery programs should be (Helm 2020; Hepburn et al. 2020).

## The Coronavirus Crisis as a Moment for Interest Groups

The expectation of huge amounts of public money being distributed at short notice brings interest groups of every shade to the scene—preferably with old wish lists on hand. Consequently, there is a big risk that recovery programs will be captured by interest groups (for an overview of the literature on regulatory capture, see Dal Bo 2006). On the one hand, climate change mitigation is put under pressure as being an “extra burden” for industries. For example, European car manufacturers have called for postponing the upcoming tightening of EU emission standards for car fleets (Topham and Harvey 2020). Some EU Member States call for stalling the EU Commission’s plan of a European green deal (Simon 2020). On the other hand, many recommend spending the public money mainly on measures that also help mitigating climate change—among them Frans Timmermans, Executive Vice-President of the European Commission (Schulz 2020), or Fatih Birol, head of the International Energy Agency (Birol 2020).

There is one thing that must not be overlooked in this politico-economic competition: public funds are still short and must be used reasonably. Otherwise ill-designed (green) subsidies can quickly turn into a part of the problem instead of being the solution. Previous “Cash for Clunkers” programs warn as an example of a misguided recovery measure. These programs were introduced in many countries after the 2009 financial crisis and provided financial incentives to trade old, less fuel-efficient cars for new, more efficient ones. Empirical analyses have shown very mixed results regarding both the economic as well as the environmental stimulus effects of these measures (Grigolon et al. 2016; Li et al. 2013; Mian and Sufi 2012). At the beginning of every discussion about (green) recovery programs it is therefore important to develop transparent and sensible criteria based on which public aid should be allocated.

## Recovery Programs Must be Climate-Proof

After the initial bail-out programs, public recovery programs to stabilize the economy are now debated politically. Certainly, this generates an unprecedented window of opportunity for structural transformation. Moreover, the distribution of public aid may also justify committing beneficiaries to public interests to a certain extent. Consequently, the currently available political degrees of freedom should be used to promote the transition of society towards sustainability. Subsidies to branches like tourism, aviation and agriculture—which are particularly hit by the crisis and are lagging behind in terms of sustainability—should be paid conditional on meeting minimum environmental standards. New investments into long-lived, fossil-fueled assets must be avoided. A recovery program cannot only be about re-establishing the status quo ante by assigning large public funds, possibly creating new barriers for sustainability transitions. In this respect, it makes sense to implement recovery programs that are in line with the objective of mitigating climate change—as called for by many at the moment. However, such green recovery programs must not be arbitrary.

## Broadband Green Subsidy Programs are Not the Solution

Green recovery programs must go beyond green subsidies. First of all, it is also important to reduce unnecessary barriers for green investments, for example by revising legal constraints to the expansion of renewable energies like solar photovoltaics of wind power. Moreover, any green recovery program can only effectively and efficiently spur decarbonization if it combines with a carbon price and the abolition of environmentally harmful subsidies. The direction of recovery must be crystal-clear. Otherwise green subsidies risk being ineffective and costly approaches to mitigating climate change (Kalkuhl et al. 2013; Palmer and Burtraw 2005), while imposing additional burdens on public budgets and reducing political degrees of freedom in the future. For subsidies to be economically justified, they need to meet clear criteria.

## Green Stimuli Must Help Stabilize the Economy

For green recovery programs to succeed in the competition for public funds with other important policy fields (such as health or digitalization), they must help stabilize the economy. Moreover, policy-makers need to be aware that some of the currently observed economic problems might even resolve without any government aid. It can expected, for example, that global supply chains will resume and that people will catch up on purchasing durable goods like cars, at least partly. It is exactly (the maintenance of) environmental regulation that may help steer this consumption towards more sustainable modes.

Government interventions must take effect where permanent disruptions are looming. One example: Innovative green business models may particularly be at risk if banks limit loans in the presence of the current uncertainties (Lehmann and Söderholm 2018). In this case, government loans may provide direct assistance. In contrast, attempts to lower prices for goods and services—e.g., for cars (VAT reduction, purchase premiums) or electricity (reduction of energy levies)—are rather inappropriate means to stabilize the economy. Such measures fail to address the actual sources of insufficient investments or reduced purchasing power, and are therefore inefficient ways of spending public budgets. Furthermore, it is unclear whether and to what extent such discounts will be passed through to final consumers by market prices (Peltzman 2000).

## Green Stimuli Must be Checked for Mitigation Potential and Maturity

Green recovery programs should focus on government interventions that would also have been economically reasonable without the COVID-19 crisis—for example, to correct market failures next to the CO_2_ externality (Bennear and Stavins 2005; Fischer and Newell 2008; Lehmann 2012)—and that have the highest priority for climate policy. Moreover, those measures should be implemented for which rational concepts have been drafted already and that can be realized promptly. Positive examples of such “no-regret measures” can be found in the transport sector, for instance. This sector is severely lagging behind in terms of climate change mitigation, and economic rationales for public expenditures exist at least partly (Briggs et al. 2015; Low and Astle 2009). In addition to that, numerous actors have already developed elaborated programs of measures. Those measures that can be implemented quickly, should now be launched—for instance to electrify the transport sector or to strengthen public transport.

## Polluters Should Contribute to Funding Green Recovery Programs

(Green) recovery programs must not only address the expenditure side. A currently still disregarded issue is the question how the required billions of Euro could be raised. Public expenditures for a green recovery program should at least partly be funded by polluters by implementing a carbon price and abandoning ecological harmful subsidies. Such policies internalizing environmental costs would not be an extra sacrifice—but rather part of the solution both for revenue problems and for the redirection towards sustainability (for a review of the double-divident hypothesis, see Goulder 2013).

## Conclusion: Combine Economic Stabilization, Mitigation of Climate Change, and Revenue Raising

The coronavirus crisis has opened up a window of opportunity for transformation. This should be used without getting off the regulatory track. Green recovery programs must not be reduced to a mere competition for green subsidies. Abandoning barriers to green investments and imposing a carbon price are equally important. Where economically sensible, green subsidies should contribute both to stabilizing the economy and mitigating climate change. Moreover, smart green recovery programs may contribute to raising revenues for the additionally necessary public expenditures.
